# Evaluating multimodal ChatGPT for emergency decision-making of ocular trauma cases

**DOI:** 10.3389/fcell.2025.1564054

**Published:** 2025-03-27

**Authors:** Jiezheng Xue, Zhouqian Wang, Nuo Chen, Yue Wu, Zhaomeng Shen, Yi Shao, Heding Zhou, Zhongwen Li

**Affiliations:** ^1^ Ningbo Key Laboratory of Medical Research on Blinding Eye Diseases, Ningbo Eye Institute, Ningbo Eye Hospital, Wenzhou Medical University, Ningbo, China; ^2^ Department of Ophthalmology, Shanghai General Hospital, Shanghai Jiao Tong University School of Medicine, National Clinical Research Center for Eye Disease, Shanghai, China; ^3^ National Clinical Research Center for Ocular Diseases, Eye Hospital, Wenzhou Medical University, Wenzhou, China

**Keywords:** artificial intelligence, large language models, ChatGPT, ocular trauma, emergency

## Abstract

**Purpose:**

This study aimed to evaluate the potential of ChatGPT in diagnosing ocular trauma cases in emergency settings and determining the necessity for surgical intervention.

**Methods:**

This retrospective observational study analyzed 52 ocular trauma cases from Ningbo Eye Hospital. Each case was input into GPT-3.5 turbo and GPT-4.0 turbo in Chinese and English. Ocular surface photographs were independently incorporated into the input to assess ChatGPT’s multimodal performance. Six senior ophthalmologists evaluated the image descriptions generated by GPT-4.0 turbo.

**Results:**

With text-only input, the diagnostic accuracy rate was 80.77%–88.46% with GPT-3.5 turbo and 94.23%–98.08% with GPT-4.0 turbo. After replacing examination information with photography, GPT-4.0 turbo’s diagnostic accuracy rate decreased to 63.46%. In the image understanding evaluation, the mean completeness scores attained 3.59 ± 0.94 to 3.69 ± 0.90. The mean correctness scores attained 3.21 ± 1.04 to 3.38 ± 1.00.

**Conclusion:**

This study demonstrates ChatGPT has the potential to help emergency physicians assess and triage ocular trauma patients properly and timely. However, its ability in clinical image understanding needs to be further improved.

## Introduction

Ocular trauma represents one of the most prevalent injuries encountered in emergency departments and stands as the leading cause of non-congenital monocular blindness in both pediatric and adult populations (Messman, 2015; Gervasio et al., 2015). The etiology of emergency eye injuries is diverse, encompassing foreign body intrusion, falls, physical assaults, thermal or chemical burns, and motor vehicle accidents. The acute onset, complex nature, and individualized presentations of ocular trauma in emergency settings underscore the critical necessity for rapid and specialized intervention. Healthcare providers face the challenging task of rendering accurate diagnoses and treatment decisions within a narrow time frame, as delays or errors in management can lead to potentially life-threatening outcomes (Yan, 2019; Iserson and Moskop, 2007).

Numerous studies have proposed sight-preserving protocols for managing routine and severe ocular injuries to facilitate efficient triage of ocular trauma patients and ensure prompt, appropriate treatment (Lindfield and Das-Bhaumik, 2009; Khare et al., 2008). The practical implementation of these protocols necessitates the expertise of ophthalmologists. However, a significant challenge arises from the scarcity of specialized ophthalmologists in emergency departments, particularly general hospitals. This shortage of medical expertise is severe in remote regions and developing nations, where the shortage of healthcare resources is even more pronounced (Resnikoff et al., 2012).

Large language models (LLMs), a form of generative artificial intelligence (AI), possess the capability to construct contextually appropriate and meaningful text based on provided inputs, effectively simulating human creativity and reasoning processes (Sanderson, 2023; The Lancet Digital Health, 2023). In recent years, applying LLMs in clinical research has seen a significant uptrend (Li et al., 2023; Rao et al., 2023; Obermeyer and Emanuel, 2016; Topol, 2019; Arora and Arora, 2023). ChatGPT, an LLM-based application developed by OpenAI utilizing advanced natural language processing (NLP) techniques, has emerged as one of the most promising tools for medical diagnosis, demonstrating remarkable potential in analyzing and interpreting complex medical data (Hosny et al., 2018; Patel and Lam, 2023). Its efficacy has been validated across various domains, including information extraction from text, document composition, educational applications, and assisting clinical practice (Lee et al., 2023; Will ChatGPT Transform Healthcare, 2023). ChatGPT has been subjected to extensive evaluation across multiple domains of clinical practice, demonstrating its potential in addressing disease-related inquiries, facilitating early patient triage, augmenting diagnostic processes, and formulating treatment recommendations (Berg et al., 2024; Huang et al., 2024; Barash et al., 2023; Benary et al., 2023). In ophthalmology, ChatGPT has exhibited certain potential in analyzing cases of glaucoma and retinal detachment (Carlà et al., 2024a; Carlà et al., 2024b). Despite its broad applications in various medical fields, the potential utility of ChatGPT in ophthalmic emergency care, particularly in the context of ocular trauma management, has not yet been studied.

In this study, we analyzed ChatGPT’s efficacy in diagnosing ocular trauma cases and determining the necessity for surgical intervention, utilizing clinical data recorded in emergency department settings. We evaluated the diagnostic capabilities of the advanced GPT-4.0 turbo model when presented with ophthalmic images. This investigation seeks to explore the potential of ChatGPT as a tool to alleviate the critical need for rapid, specialized decision support in ophthalmic emergencies, particularly in resource-constrained environments.

## Materials and methods

### Study design and participants

**FIGURE 1:**
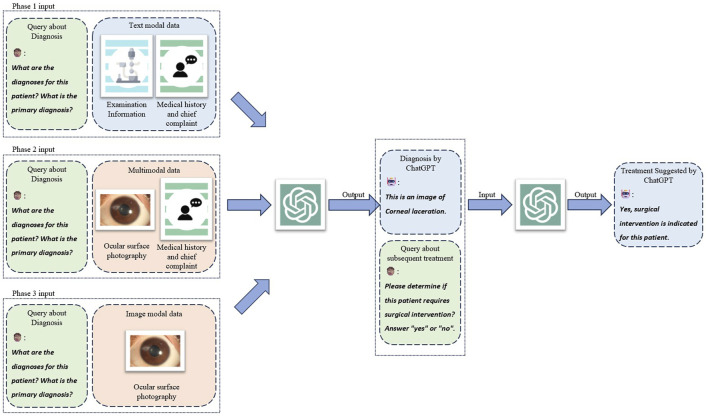
Study design and three phases of interfacing with ChatGPT. Three kinds of modal combinations were input to ChatGPT as three individual experiment phases to collect three groups of generated responses. The query of treatment suggestion was conducted on cases that ChatGPT correctly answered diagnostic query which served as the chat history as well.

**FIGURE 2:**
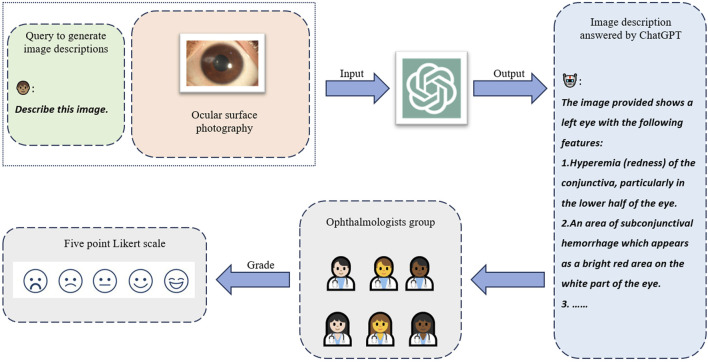
Human ophthalmologists assessing the image understanding capability of ChatGPT. ChatGPT was instructed to describe the input image in detail. The generated descriptions were delivered to six ophthalmologists to independently assess the quality in correctness and completeness with the five-point Likert scale.

This retrospective observational study evaluated the diagnostic capabilities of ChatGPT (version dated 25 January 2024; OpenAI, San Francisco, United States) in the context of ocular trauma. We assessed its performance in generating diagnoses and surgical recommendations based on medical textual records and its ability to recognize ocular trauma from ophthalmic images (Figure 1). The study design incorporated a bilingual approach, with ChatGPT responses obtained in both Chinese (CN) and English (EN) for each case. We also conducted a comparative analysis between the GPT-3.5 turbo and GPT-4.0 turbo models. Furthermore, to assess the quality and clinical relevance of image descriptions generated by GPT-4.0 turbo, we recruited a panel of six ophthalmologists with a mean (±SD) clinical experience of 9.5 (±6.9) years to independently evaluate the generated descriptions using a five-point Likert scale (Figure 2).

### Case enrollment and dataset

This study retrospectively analyzed 52 cases of patients diagnosed with ocular trauma who presented to the emergency department of Ningbo Eye Hospital between September 2022 and December 2023. Patient medical textual records were comprehensively anonymized and included medical history with chief complaints, detailed ocular examination findings, and imaging of the ocular trauma. To maintain the integrity of the evaluation process, we deliberately excluded information about the attending ophthalmologists’ assessment, treatment decisions, and examinations, thereby preventing inadvertent disclosure of diagnostic conclusions. The definitive diagnosis for each case, which served as the reference, was established by chief ophthalmologists specializing in ocular trauma. ChatGPT-generated responses were systematically collected and analyzed from 1 March to 12 March 2024.

### ChatGPT interface

To ensure the independent evaluation of ChatGPT and mitigate potential biases from chat history, we implemented a protocol wherein each case record was input into a discrete entry page. Drawing upon the demonstrated efficacy of prompt engineering in previous studies (Meskó, 2023; Savage et al., 2024), we developed a series of standardized “prompts” based on a published framework (White et al., 2023). These prompts were systematically incorporated at the initiation of each new conversation to maintain consistency and optimize the AI’s performance. The specific prompt structure and content are detailed in Supplementary Figure S1.

### Evaluation of ChatGPT’s responses based on multimodal input

The assessment of ChatGPT’s diagnostic capability was conducted in three distinct phases. In the initial phase, we employed a text-based approach, the medical history and chief complaint with eye examination information (HEI) method. This method utilized comprehensive textual input, encompassing medical history with chief complaints and detailed eye examination findings. GPT-3.5 turbo and GPT-4.0 turbo models were sequentially presented with this information in CN and EN for each case. We systematically recorded the AI-generated principal diagnosis of ocular trauma for each model in both languages. Upon confirmation of an accurate principal diagnosis, the respective ChatGPT model was further instructed to assess the necessity for surgical intervention.

In the second phase, we assessed the multimodal capabilities of GPT-4.0 turbo by integrating both textual and visual inputs. Typical examples of ocular trauma images are shown in Supplementary Figure S2. This phase was designed to simulate a real-world clinical scenario where specialized ophthalmological expertise is unavailable, and only photographic evidence and concise textual records are accessible. The primary objective was to evaluate ChatGPT’s ability to generate accurate diagnoses and appropriate treatment recommendations under these constraints. We employed the photograph with medical history and chief complaint (PCC) method, wherein the GPT-4.0 turbo was presented solely with an ocular photograph and a brief chief complaint, provided in CN and EN, respectively. The model was instructed to perform two sequential tasks: first, to describe the features of the ocular image, and second, to formulate a principal diagnosis of ocular trauma based on this visual information and the accompanying chief complaint. Subsequently, contingent upon the accuracy of the initial diagnosis, GPT-4.0 turbo was directed to assess the necessity for surgical intervention. This approach aimed to evaluate the model’s capacity for visual interpretation, diagnostic reasoning, and treatment planning in a setting that mirrors the constraints often encountered in non-specialized or resource-limited healthcare environments.

The third phase of our study focused on assessing GPT-4.0 turbo’s capability to diagnose ocular trauma solely based on visual input, without any accompanying textual information. We employed a novel approach, the photography instruct only (PIO) method, wherein all textual historical data were deliberately omitted. GPT-4.0 turbo was instructed to formulate a principal diagnosis of ocular trauma exclusively based on the patient’s ocular trauma photographs. To establish a clinically relevant benchmark for comparison, we replicated this protocol with a panel of four junior ophthalmologists, each with a mean clinical experience of 5 years. These human experts were presented with identical instructions and visual data. Their collective assessments were synthesized to derive a consensus diagnostic outcome. The conversation examples for the three methods (HEI, PCC, PIO) were displayed in Supplementary Figure S3.

### Human doctors evaluating the generated contents by ChatGPT

To further evaluate the image comprehension capabilities of ChatGPT, we assessed the descriptive content generated by GPT-4.0 turbo for ocular trauma images. A panel of six experienced ophthalmologists evaluated the AI-generated descriptions using standardized five-point Likert scales to assess both completeness and correctness. The completeness of image descriptions was rated on the following scale: 1) Severely incomplete: Lacking critical elements of the image. 2) Partially incomplete: Limited inclusion of critical elements or image details. 3) Moderately complete: Providing some necessary information. 4) Substantially complete: Capturing the majority of important information. 5) Comprehensively complete: Encompassing all important elements and additional relevant details. The correctness of image descriptions was evaluated using this scale: 1) Entirely inaccurate: Descriptions with no correct interpretations. 2) Largely inaccurate: Descriptions with limited correct interpretation and critical errors. 3) Moderately accurate: Descriptions with a generally correct image interpretation. 4) Highly accurate: Descriptions with a complete interpretation but potential minor errors. 5) Perfectly accurate: Descriptions with a flawless and complete interpretation.

### Observation indicators

This study evaluated the accuracy of ChatGPT in diagnosing ocular trauma and guiding surgical treatment under varying model configurations and language settings. The primary outcome measure was the accuracy of diagnosis, with surgical treatment deemed effective only if the diagnosis was correct. Additionally, the study assessed the completeness and correctness of image-based descriptions generated by the GPT-4.0 turbo model, as evaluated by six ophthalmologists.

### Statistical analysis

Statistical analyses were performed using R statistical software version 4.0.2. Continuous variables were presented as mean (standard deviation), while categorical data were reported as absolute frequencies (number, N) and percentages. The Wilcoxon test was used to compare continuous data, and the chi-square test was used for categorical data comparisons. Statistical significance was a two-sided p-value less than or equal to 0.05. To demonstrate the distribution and consistency of the Likert scales in two languages, we adopted the kernel density plots to visualize the results.

## Results

### Patient characteristics

This study encompassed a cohort of 52 patients presenting with ocular trauma requiring emergency care. The predominant injury type was open-globe injuries, accounting for 43 cases (82.69%), while the remaining 9 cases (17.31%) were classified as closed-globe injuries. The patient demographics were characterized by a median age of 43.5 years (7–74 years) and a notable male predominance (n = 45, 86.54%). Concerning clinical management, most patients (n = 46, 88.46%) underwent surgical intervention, while a small subset (n = 6, 11.54%) were managed conservatively. A detailed overview of the baseline patient characteristics and injury classifications is presented in Table 1.

**TABLE 1:** Demographic characteristics, standard diagnosis, and actual surgical acceptance of patients with eye trauma.

Variable	Participants, No. (%) (N = 52)
Age, median (IQR) [range], y	43.5 (31–52) [7–74]
Sex	
Female	7 (13.46%)
Male	45 (86.54%)
Diagnostic category	
Rupture of the eyeball	18 (34.62%)
Corneal penetrating injury	24 (46.15%)
Blunt contusion	6 (11.54%)
Corneal Foreign Body	2 (3.85%)
Corneal Laceration	2 (3.85%)
Surgical treatment	
Yes	46 (88.46%)
No	6 (11.54%)

IQR, interquartile range.

### Diagnostic accuracy of GPT-3.5 turbo and GPT-4.0 turbo models

The diagnostic responses generated by ChatGPT for the enrolled cases are detailed in Supplementary Table S1. In the initial phase, which involved analysis of medical textual records and ophthalmologic examination findings, the GPT-3.5 turbo model demonstrated diagnostic accuracies of 80.77% and 88.46% for Chinese and English inputs, respectively (GPT-3.5-HEI-CN vs. GPT-3.5-HEI-EN, p = 0.277). The GPT-4.0 turbo model exhibited superior performance, achieving diagnostic accuracies of 98.08% for Chinese and 94.23% for English inputs (GPT-4.0-HEI-CN vs. GPT-4.0-HEI-EN, p = 0.610). In the second phase, where ophthalmologic examination findings were omitted but ocular images were included, the GPT-4.0 turbo model’s performance declined to 63.46% accuracy for both languages (GPT-4.0-PCC-CN vs. GPT-4.0-PCC-EN, p = 1.000). The third phase, utilizing solely ocular images without any textual data, resulted in further reduced accuracies: 26.92% for Chinese and 32.69% for English inputs with GPT-4.0 turbo (GPT-4.0-PIO-CN vs. GPT-4.0-PIO-EN, p = 1.000). Notably, under the same image-only conditions, a control group of ophthalmologists achieved a substantially higher diagnostic accuracy of 80.29%.

**FIGURE 3:**
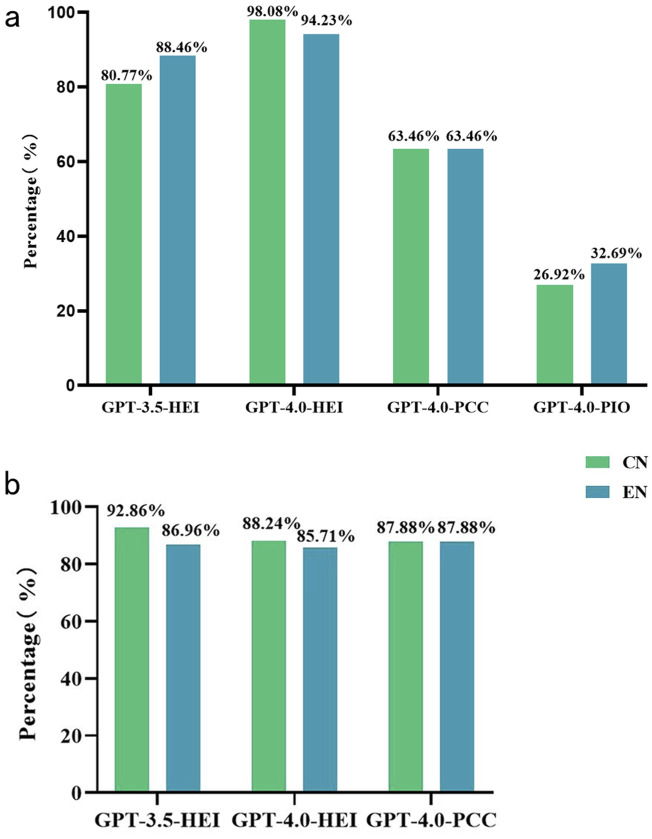
The performance of ChatGPT in diagnosing and providing treatment suggestions for ocular trauma with different data models. **(a)** Diagnostic accuracy of GPT-3.5 turbo and GPT-4.0 turbo in Chinese and English with different input models. **(b)** Accuracy of determining the need for surgical treatment (only for patients with a correct ChatGPT diagnosis). CN Chinese, EN English, HEI medical history and chief complaint with eye examination information, PCC photograph with medical history and chief complaint, PIO photography instruct only, GPT Generative Pre-trained Transformer.

In the comparative model performance analysis, GPT-4.0-HEI-CN demonstrated statistically significant superiority over GPT-3.5-HEI-CN in diagnostic accuracy for text-only queries during the first phase (p = 0.004). Interestingly, no significant difference was observed between GPT-3.5-HEI-EN and GPT-4.0-HEI-EN for English language inputs (p = 0.485). For the multimodal capabilities of GPT-4.0 turbo, the text-based GPT-4.0-HEI significantly outperformed the image-augmented GPT-4.0-PCC in both Chinese and English communications (p < 0.001). This suggests a potential limitation in the model’s ability to integrate visual data with textual information effectively. A comparison of diagnostic accuracies across all three phases of the study is visually represented in Figure 3a, illustrating the performance variations under different input modalities and language conditions.

### Accuracy of ChatGPT in surgical recommendations

The surgical recommendations generated by ChatGPT for the enrolled cases are detailed in Supplementary Table S2. In the analysis of language impact on surgical judgment in Figure 3b, the GPT-3.5 turbo model demonstrated no statistically significant difference between Chinese and English inputs (GPT-3.5-HEI-CN 92.86% vs. GPT-3.5-HEI-EN 86.96%, p = 0.575). The GPT-4.0 turbo model exhibited similar language-independent performance, with comparable accuracies in Chinese (GPT-4.0-HEI-CN 88.24%) and English (GPT-4.0-HEI-EN 85.71%, p = 0.708). Furthermore, no statistically significant differences were observed between GPT-3.5-HEI and GPT-4.0-HEI models in either Chinese (p = 0.691) or English (p = 0.860) communications. Regarding multimodal performance, the GPT-4.0-PCC model, which incorporated ocular images but lacked examination text, showed identical accuracy rates in both languages (GPT-4.0-PCC-CN 87.88% vs. GPT-4.0-PCC-EN 87.88%, p = 1.000). Notably, the performance of GPT-4.0 turbo appeared equivalent whether provided with examination text (HEI) or ocular images (PCC) in both Chinese (GPT-4.0-HEI-CN vs. GPT-4.0-PCC-CN, p = 1.000) and English (GPT-4.0-HEI-EN vs. GPT-4.0-PCC-EN, p = 1.000). This suggests that, for surgical recommendation tasks, the model’s performance was not significantly influenced by the modality of input data. Given that appropriate treatment recommendations rely on accurate diagnoses, GPT-4.0-PIO was excluded from this phase due to its poor diagnostic performance.

### Evaluation of GPT-4.0 turbo in image descriptions

**FIGURE 4:**
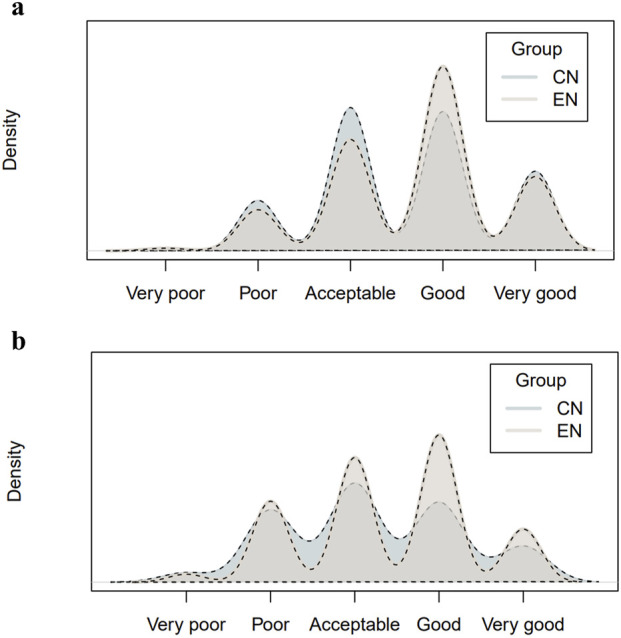
Distribution of the completeness and correctness of the image descriptions answered by GPT-4.0 turbo. Density plots of the ratings distribution from six independent board-certified ophthalmologists are shown. Plot **(a)** shows completeness ratings; Plot **(b)** shows correctness ratings.

The GPT-4.0-PCC model demonstrated comparable levels of completeness in image descriptions for both Chinese (CN) and English (EN) languages. The mean completeness scores were 3.59 ± 0.94 and 3.69 ± 0.90 for GPT-4.0-PCC-CN and GPT-4.0-PCC-EN, respectively (n = 312, Wilcoxon = 45,389, p = 0.125). However, GPT-4.0-PCC exhibited significantly better performance in English than in Chinese regarding image description correctness. The mean correctness scores were 3.21 ± 1.04 for GPT-4.0-PCC-CN and 3.38 ± 1.00 for GPT-4.0-PCC-EN (n = 312, Wilcoxon = 43,942, p = 0.029) (Figure 4).

## Discussion

In emergency departments, accurate and timely diagnosis of ocular trauma is important to improve the patient’s visual prognosis. This comparative study explored ChatGPT’s performance in diagnosing ocular trauma and providing surgical recommendations in real-world emergency cases, utilizing bilingual and multimodal data. We evaluated two state-of-the-art large language models proposed by OpenAI: GPT-3.5 turbo and GPT-4.0 turbo. Both Chinese and English communications were compared. Our study revealed that the GPT-4.0-HEI-CN method achieved a diagnostic accuracy of 98.08%. Besides, the choice of interaction language did not significantly affect ChatGPT’s performance, regardless of whether GPT-3.5 turbo or GPT-4.0 turbo was used, highlighting ChatGPT’s compatible bilingual capabilities. We also observed that GPT-4.0 turbo outperformed GPT-3.5 turbo when processing Chinese medical textual records and examination findings, providing valuable insights for model and language selection in future research. To further explore ChatGPT’s potential in surgical decision-making, we instructed the model to give recommendations on the necessity of surgical treatment after confirming the diagnosis. The GPT-3.5-HEI-CN method achieved an accuracy of 92.86% in surgical judgment, with no significant differences observed between models or languages.

To interpret the possible reasons for the performance degradation in diagnostic accuracy (from 94.23% to 98.08%–63.46%) when examination findings were replaced with an ocular surface image, we enlisted six ophthalmologists to evaluate the quality of ChatGPT-generated descriptions of the input ocular surface photographs. The results revealed that ChatGPT fell short of providing complete and accurate descriptions of images. This finding suggests that the multimodal capabilities of Artificial General Intelligence (AGI) are still in their nascent stages and are currently not sufficiently advanced for reliable application in medical practices. Currently, some researches revealed that LLMs possess certain potential in analyzing OCT and OCTA images, while their image understanding capabilities still require further optimization, with the expectation that it can be utilized in future real-world scenarios (Carlà et al., 2024c).

In real-world emergency departments, particularly in remote areas, the absence of specialized ophthalmologists can significantly delay patients with ocular trauma receiving immediate emergency care. While ChatGPT demonstrated promising diagnostic accuracy when provided with comprehensive medical histories containing detailed examination information, its performance deteriorated substantially without such data, even when supplemented with additional image input. Notably, the diagnostic accuracy plummeted to 26.92%–32.69% when images were the only valid data available to ChatGPT, whereas junior ophthalmologists achieved a diagnostic accuracy of 80.29% under the same conditions. These observations underscore ChatGPT’s limited capability in multimodal tasks related to ocular trauma. Given these findings, it is evident that ChatGPT’s appropriate clinical role should be that of a copilot, assisting human ophthalmologists rather than attempting to replace them. This collaborative approach leverages the strengths of both AI and human expertise to optimize patient care in ocular trauma cases.

This study has several limitations. First, using two-dimensional images potentially underestimates ChatGPT’s capabilities in spatial awareness. For instance, ChatGPT misclassified corneal wounds as corneal foreign bodies in some cases (Supplementary Table S3), likely due to the similarity in appearance between irregular corneal wounds and foreign bodies such as glass fragments. This challenge, also reported by Li et al. in classic deep learning models (Li et al., 2021), could lead to significant diagnostic errors. Second, using LLMs presents inherent challenges, including the potential for misleading or biased outputs, lack of transparency, and limited access to up-to-date knowledge. While the frequency of misleading responses decreases with model evolution (King, 2023; Radford et al., 2018), synthetic answers containing misinformation could still lead to dangerous medical decisions and potential patient harm. Future research will focus on evaluating the performance of multimodal large models using more diverse datasets, further exploring the possible applications of AGI in ophthalmology.

## Conclusion

This study evaluated ChatGPT’s capability to diagnose ocular trauma and recommend surgical interventions based on multimodal inputs, including textual and image data. Our findings suggest that ChatGPT has the potential to serve as a valuable tool for ophthalmologists, acting as a copilot in their decision-making process, although it is not yet ready to serve patients independently. In addition, ChatGPT demonstrated proficiency in analyzing detailed textual information to make informed emergency decisions for ocular trauma cases. However, its ability to analyze ocular photographs remains insufficient for clinical application. The development of AGI requires further refinement before it can be directly implemented in clinical settings. These results underscore the promise of AI in ophthalmology while highlighting the ongoing need for human expertise in patient care.

## Data Availability

The original contributions presented in the study are included in the article/Supplementary Material, further inquiries can be directed to the corresponding authors.
