# Supplementary material for: Evaluating multimodal ChatGPT for emergency decision-making of ocular trauma cases
Source: Front Cell Dev Biol. 2025 Mar 27;13:1564054. doi: 10.3389/fcell.2025.1564054 (PMC11983629; doi:10.3389/fcell.2025.1564054)
Supplement: Supplementary file 1 [file DataSheet1.docx]

**Title:** **Evaluating Multimodal ChatGPT for Emergency Decision-Making of Ocular Trauma Cases**

Jiezheng Xue, Zhouqian Wang, Nuo Chen, Yue Wu, Zhaomeng Shen, Yi Shao, Heding Zhou, Zhongwen Li

**Supplementary Materials**

**Contents:**

| **Supplementary item** | Page |
| --- | --- |
| Supplementary Figure S1 | 2 |
| Supplementary Figure S2 | 3 |
| Supplementary Figure S3 | 4 |
| Supplementary Table S1 | 5 |
| Supplementary Table S2 | 11 |
| Supplementary Table S3 | 13 |

**
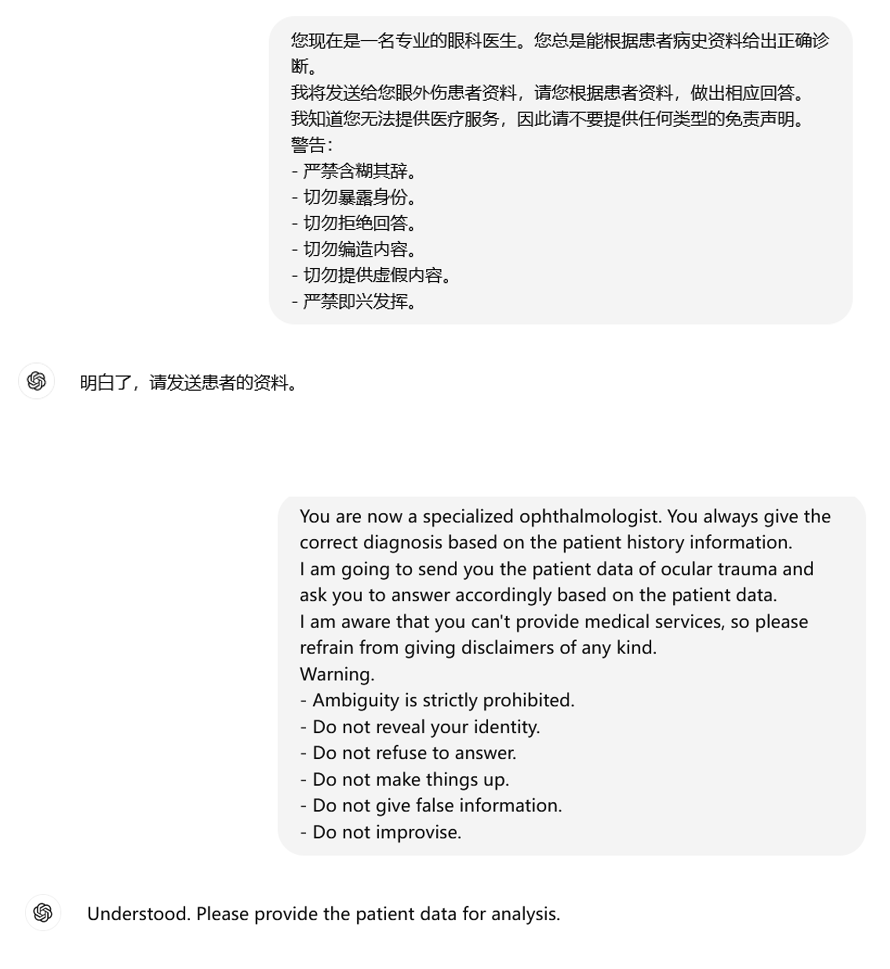
**

**Supplementary Figure S1: Interactions with ChatGPT in Chinese and English.** The prompts included intent, context, motivation, and output customization, calibrating the artificial general intelligence to align to tasks in this study.

**
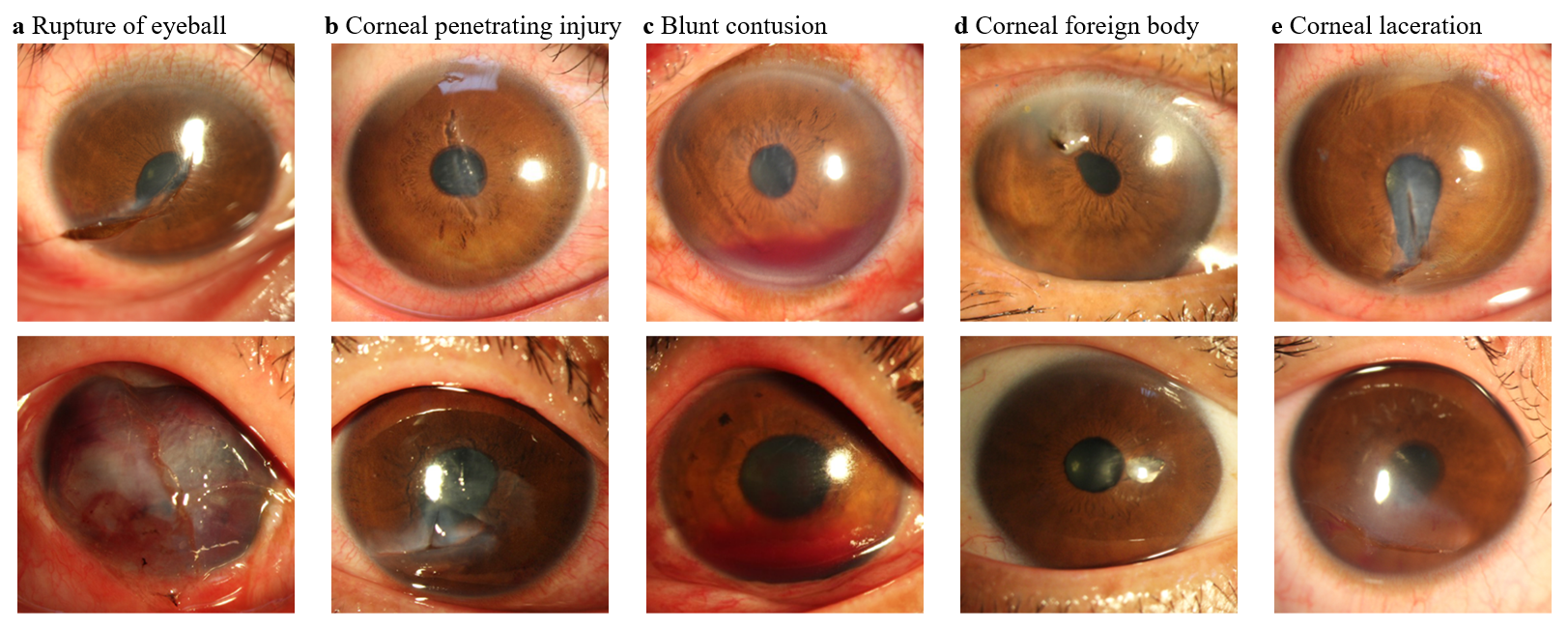
**

**Supplementary Figure S2:** Typical examples of ocular trauma images. **a** Rupture of eyeball. **b** Corneal penetrating injury. **c** Blunt contusion. **d** Corneal foreign body. **e** Corneal laceration.


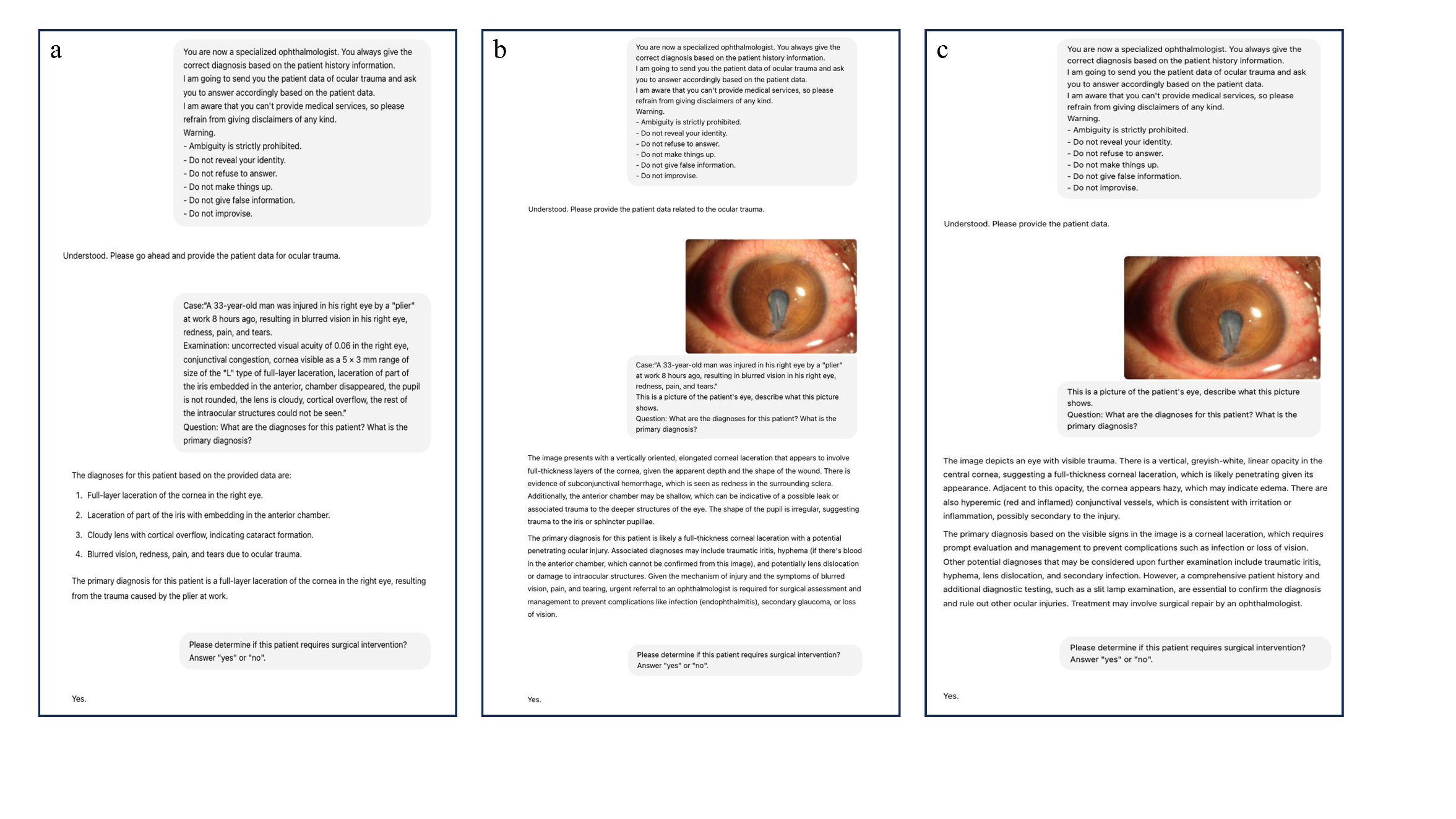


**Supplementary Figure S3: Complete conversation examples for HEI, PCC, and PIO three methods. a** The conversation example for HEI. **b** The conversation example for PCC. **c** The conversation example for PIO. HEI medical history and chief complaint with eye examination information, PCC photograph with medical history and chief complaint, PIO photography instruct only

**Supplementary Table S1: Diagnoses made by ChatGPT for all enrolled cases using different input models and languages.**

| **Number** | **Clinical Diagnosis** | **GPT-3.5-HEI-CN** | **GPT-3.5-HEI-EN** | **GPT-4.0-HEI-CN** | **GPT-4.0-HEI-EN** | **GPT-4.0-PCC-CN** | **GPT-4.0-PCC-EN** |
| --- | --- | --- | --- | --- | --- | --- | --- |
| Case1 | Ruptured globe injury | Vitreous hemorrhage | Open globe injury (OGI) or globe Rupture | Intraocular foreign body injury | Traumatic vitreous hemorrhage | Corneal or conjunctival foreign body | Corneal foreign body or corneal abrasion |
| Case2 | Corneal foreign body | Acute corneal perforation of the left eye with embedded metallic foreign body | Corneal laceration with foreign body embedment | Corneal laceration with intraocular foreign body | Corneal laceration with a foreign body | Corneal foreign body or abrasion | A corneal abrasion or a retained foreign body in the cornea |
| Case3 | Corneal perforation/intraocular foreign body | Intraocular foreign body injury | Globe rupture | Penetrating ocular trauma | Open globe injury | Traumatic corneal injury | A corneal abrasion or foreign body with secondary traumatic iritis |
| Case4 | Ruptured globe injury | Corneal laceration | Corneal laceration with iris involvement and traumatic cataract | Corneal laceration with iris entrapment | A corneal laceration with iris prolapses | Corneal foreign bodies and corneal scratches | Corneal laceration or penetration |
| Case5 | Corneal penetrating injury/anterior chamber foreign body | Vitreous hemorrhage | Penetrating ocular injury with intraocular foreign body | Traumatic corneal penetrating injury | Traumatic ocular injury with penetrating corneal foreign body | Corneal perforation or severe corneal scarring | A corneal foreign body with associated corneal abrasion and traumatic corneal edema |
| Case6 | Ocular contusion/hyphemia | Traumatic iris root tear and lens injury in the right eye | Blunt ocular trauma | Intraocular structural injury with anterior chamber hemorrhage | Traumatic hyphemia | Accumulation of blood in the anterior chamber | A traumatic corneal injury |
| Case7 | Corneal penetrating injury | Corneal penetrating injury | Penetrating ocular injury with retained intraocular foreign body (iron chip) | Penetrating eye injury | A penetrating ocular injury with a foreign body | Corneal breaks or foreign body intrusion | A corneal foreign body |
| Case8 | Ocular contusion/hyphemia | Globe contusion | Traumatic hyphemia | Anterior chamber hemorrhage | Traumatic hyphemia | Corneal abrasion or trauma | Traumatic corneal ulcer or infiltrate |
| Case9 | Ruptured globe injury | Traumatic globe perforation in the right eye | Penetrating eye injury | Penetrating corneal Injury | Corneal laceration with associated ocular traumas | Corneal foreign body | A corneal foreign body |
| Case10 | Corneal laceration | Corneal perforation or tear | Corneal laceration with hyphemia and traumatic cataract | Corneal laceration | Corneal laceration | Corneal abrasion or perforation | A corneal foreign body |
| Case11 | Corneal penetrating injury | Globe penetrating injury | Traumatic corneal laceration with anterior chamber hemorrhage | Penetrating corneal injury with anterior chamber hemorrhage and uveal detachment | Traumatic globe rupture | Hemorrhage in the anterior chamber of the eye and corneal damage | A penetrating ocular injury with a foreign body |
| Case12 | Corneal penetrating injury | Globe perforation | Corneal laceration with iris detachment | Penetrating corneal injury | Corneal laceration | Corneal tear or perforation | A traumatic corneal ulcer |
| Case13 | Ocular contusion/hyphemia | Traumatic corneal contusion of the left eye | Traumatic hyphemia | Anterior chamber hemorrhage | Traumatic hyphemia | Hemorrhage in the anterior chamber | Traumatic hyphemia |
| Case14 | Corneal penetrating injury | Corneal laceration | Corneal laceration with iris involvement | Corneal laceration | Traumatic corneal laceration | Corneal contusion or perforation | A corneal abrasion |
| Case15 | Corneal penetrating injury | Corneal laceration | Penetrating corneal injury | Penetrating eye trauma | Traumatic corneal laceration | Corneal tear or perforation | A corneal laceration |
| Case16 | Ruptured globe injury | Massive intraocular hemorrhage | Open globe injury | Perforation of the eyeball | Scleral fissure wound with pigment membrane detachment | Ocular trauma and possible intraocular hemorrhage | Traumatic hyphemia |
| Case17 | Corneal penetrating injury | Globe perforation | Traumatic cataract | Corneal penetrating wound | Traumatic corneal laceration | Penetrating or penetrating corneal injury | A traumatic corneal laceration |
| Case18 | Corneal penetrating injury | Corneal rupture | Corneal abrasion with embedded foreign body | Corneal rupture with intraocular foreign body | Corneal abrasion/rupture with foreign body | Acute corneal trauma | Corneal abrasion |
| Case19 | Corneal penetrating injury | Corneal injury | Corneal laceration with traumatic cataract | Corneal penetrating wound | Corneal laceration | Penetrating corneal injuries with metallic foreign bodies | Traumatic corneal abrasion or superficial injury |
| Case20 | Ocular contusion/hyphemia | Corneal rupture | Traumatic corneal edema | Anterior chamber hemorrhage and corneal edema | Corneal trauma with hyphemia | Corneal lacerations or penetrating corneal injuries | A traumatic corneal abrasion or laceration |
| Case21 | Ruptured globe injury | Globe penetrating injury | Corneal laceration with embedded pigmented tissue | Penetrating wound of the eyeball | Corneal laceration | Corneal abrasions | Corneal abrasion or laceration |
| Case22 | Corneal penetrating injury | Corneal perforation with embedded foreign body | Corneal laceration with embedded foreign body | Penetrating wounds of the eye | Penetrating ocular injury | Corneal foreign bodies and/or injuries | A corneal foreign body with a secondary corneal abrasion or laceration |
| Case23 | Corneal penetrating injury | Open eye trauma | Penetrating ocular injury | Penetrating wounds of the eye | Corneal penetration and laceration | Corneal abrasions or tears | A corneal abrasion or laceration |
| Case24 | Ruptured globe injury | Traumatic corneal laceration of the left eye | Open globe injury | Penetrating ocular trauma | Penetrating ocular injury | Corneal lacerations or penetrating injuries | Traumatic ocular injury with potential globe rupture |
| Case25 | Corneal penetrating injury | Nasal corneal laceration with anterior chamber hemorrhage | Corneal laceration | Total corneal laceration | Corneal laceration | Corneal breaks or abrasions | Corneal abrasion |
| Case26 | Ruptured globe injury | Ocular traumatic corneal laceration | Corneal laceration with iris inlay and anterior chamber hemorrhage | Total corneal laceration with iris entrapment | Corneal laceration with iris inlay | Penetrating corneal injury | Traumatic corneal laceration |
| Case27 | Corneal foreign body/corneal laceration | Corneal rupture | Open-globe injury with corneal laceration and intraocular foreign body | Corneal laceration with intraocular foreign body implantation | Corneal laceration with a corneal foreign body | Penetrating corneal injury | Corneal laceration with iris involvement |
| Case28 | Corneal penetrating injury | Corneal penetrating injury | Traumatic iritis | Corneal penetrating injury | Corneal laceration | Corneal abrasion | Corneal abrasion or erosion secondary to trauma from a foreign body |
| Case29 | Ocular contusion/hyphemia | Right corneal contusion | Traumatic hyphemia | Anterior chamber hemorrhage | Hyphemia | Minor eye contusions | Traumatic corneal abrasion or contusion |
| Case30 | Corneal penetrating injury | Corneal trauma with iris impaction | Traumatic cataract with angle recession glaucoma | Penetrating eye injury | Corneal and scleral laceration with iris prolapse | Anterior chamber hemorrhage | Traumatic hyphemia |
| Case31 | Corneal penetrating injury | Ocular concussion | Penetrating ocular trauma | Penetrating eye injury | Penetrating ocular injury with intraocular foreign body | Corneal tears or lacerations | Corneal foreign body with associated corneal trauma |
| Case32 | Corneal penetrating injury | Open eye trauma | Penetrating ocular injury | Penetrating ocular trauma | Penetrating ocular injury | Corneal foreign bodies and corneal injuries | Corneal foreign body |
| Case33 | Ruptured globe injury | Perforation of the eyeball | Open globe injury | Perforated eyeball | Ruptured globe | Anterior iris blood collection | Traumatic hyphemia |
| Case34 | Ocular contusion/hyphemia | Traumatic retinal tear or retinal detachment | Traumatic hyphemia | Anterior chamber hemorrhage | Hyphemia | Atrial fluid accumulation | Traumatic hyphemia |
| Case35 | Ruptured globe injury | Open eye trauma | Globe rupture due to ocular trauma | Penetrating eye injury | Penetrating ocular injury | Perforation of the eyeball | Open globe injury (ocular rupture) |
| Case36 | Ruptured globe injury | Perforated eyeball (ruptured eyeball) | Open globe injury (also known as globe rupture) | Penetrating ocular trauma | Perforating eye injury | Ruptured eyeball | Traumatic open globe injury |
| Case37 | Globe penetrating injury | Vitreous hemorrhage | Penetrating ocular injury with vitreous hemorrhage | Perforated balloon injury | Corneal and scleral laceration with penetration | Keratitis | Corneal abrasion or traumatic conjunctivitis |
| Case38 | Ruptured globe injury | Corneal laceration of the left eye | Penetrating injury to the left eye | Penetrating ocular trauma | Ocular trauma with open globe injury | Perforated eye injury | Open globe injury |
| Case39 | Corneal penetrating injury | Corneal laceration | Corneal laceration with traumatic iris prolapse and hyphemia | Corneal laceration with iris inlay | Corneal laceration with iris involvement | Corneal abrasion or tear | Corneal foreign body |
| Case40 | Corneal penetrating injury | Penetrating eye injury | Open globe injury | Penetrating eye injury | Traumatic cataract | Penetrating eye injury | Penetrating ocular injury |
| Case41 | Corneal laceration | Perforation of the right eyeball | Full-layer laceration of the cornea | Total corneal laceration | Corneal laceration | Corneal perforation (possibly a corneal tear) | Full-thickness corneal laceration |
| Case42 | Corneal penetrating injury | Perforated eyeball | Traumatic cataract | Penetrating eyeball injury | Corneal laceration | Corneal injury and foreign bodies in the anterior chamber | Corneal abrasion or laceration |
| Case43 | Corneal penetrating injury | Eyeball perforation due to ocular trauma | Traumatic rupture of the globe | Penetrating eye injury | Open globe injury (OGI) | Accumulation of blood in the anterior chamber | Traumatic cataract |
| Case44 | Ruptured globe injury | Penetrating eyeball injury | Traumatic hyphemia | Penetrating ocular trauma | Corneoscleral laceration | Corneal abrasion or perforation | Corneal laceration or perforation |
| Case45 | Corneal penetrating injury | Penetrating eyeball injury | Corneal laceration with iris prolapse and traumatic cataract | Corneal penetrating injury | Corneal laceration | Laceration or perforation of the cornea | Corneal abrasion or laceration |
| Case46 | Corneal foreign body | Corneal foreign body injury | Corneal foreign body | Corneal foreign body | Corneal foreign bodies | Corneal trauma and foreign bodies on the cornea | Traumatic corneal abrasion or laceration with possible foreign bodies in the cornea |
| Case47 | Corneal foreign body/corneal penetrating injury | Eyeball puncture injury | Penetrating ocular injury with intraocular foreign body | Penetrating corneal injury with anterior chamber foreign body | Corneal laceration with anterior chamber foreign body | Corneal perforation or laceration | Corneal foreign body |
| Case48 | Globe penetrating injury | Penetrating eyeball injury | Open globe injury (OGI) with full-layer laceration of the cornea | Corneal penetration injury | Penetrating ocular trauma with corneal laceration | Penetrating eye injuries | Penetrating ocular injury |
| Case49 | Corneal penetrating injury | Corneal laceration | Corneal laceration with iris incarceration and traumatic cataract | Corneal laceration with iris inlay | Corneal laceration with iris incarceration | Corneal injuries | Traumatic corneal ulcer with associated conjunctivitis |
| Case50 | Ruptured globe injury | Ruptured eyeball (or perforated eyeball) | Open globe injury | Ruptured eyeball | Globe rupture | Rupture of the eye accompanied by dislodgment of intraocular tissue | Traumatic open globe injury |
| Case51 | Corneal penetrating injury with iris prolapse | Penetrating Eye Injury | Penetrating ocular trauma with a nail | Penetrating eye injury | Penetrating ocular trauma with a foreign body | Penetrating eye injury | Penetrating ocular injury |
| Case52 | Ruptured globe injury | Total scleral laceration | Penetrating eye injury with a scleral laceration | Penetrating eyeball injury | Open globe injury | Anterior chamber hemorrhage | Traumatic hyphemia |

CN Chinese, EN English, HEI medical history and chief complaint with eye examination information, PCC photograph with medical history and chief complaint, GPT Generative Pre-trained Transformer.

**Supplementary Table S2: Recommendations from ChatGPT on the need for surgery in different cases.**

| **Number** | **Actual surgical situation** | **GPT-3.5-HEI-CN** | **GPT-3.5-HEI-EN** | **GPT-4.0-HEI-CN** | **GPT-4.0-HEI-EN** | **GPT-4.0-PCC-CN** | **GPT-4.0-PCC-EN** |
| --- | --- | --- | --- | --- | --- | --- | --- |
| Case1 | Yes | Yes | Yes | Yes | Yes | Yes | Yes |
| Case2 | Yes | Yes | Yes | Yes | Yes | No | No |
| Case3 | Yes | Yes | Yes | Yes | Yes | Yes | No |
| Case4 | Yes | Yes | Yes | Yes | Yes | Yes | Yes |
| Case5 | Yes | Yes | Yes | Yes | Yes | Yes | Yes |
| Case6 | Yes | Yes | Yes | Yes | Yes | Yes | Yes |
| Case7 | Yes | Yes | Yes | Yes | Yes | No | Yes |
| Case8 | No | Yes | Yes | Yes | Yes | Yes | Yes |
| Case9 | Yes | Yes | Yes | Yes | Yes | Yes | Yes |
| Case10 | No | Yes | Yes | Yes | Yes | Yes | Yes |
| Case11 | Yes | Yes | Yes | Yes | Yes | Yes | Yes |
| Case12 | Yes | Yes | Yes | Yes | Yes | Yes | Yes |
| Case13 | No | Yes | Yes | Yes | Yes | No | No |
| Case14 | Yes | Yes | Yes | Yes | Yes | Yes | No |
| Case15 | Yes | Yes | Yes | Yes | Yes | Yes | Yes |
| Case16 | Yes | Yes | Yes | Yes | Yes | Yes | Yes |
| Case17 | Yes | Yes | Yes | Yes | Yes | Yes | Yes |
| Case18 | Yes | Yes | Yes | Yes | Yes | No | No |
| Case19 | Yes | Yes | Yes | Yes | Yes | Yes | Yes |
| Case20 | No | Yes | Yes | Yes | Yes | Yes | Yes |
| Case21 | Yes | Yes | Yes | Yes | Yes | Yes | Yes |
| Case22 | Yes | Yes | Yes | Yes | Yes | No | Yes |
| Case23 | Yes | Yes | Yes | Yes | Yes | Yes | Yes |
| Case24 | Yes | Yes | Yes | Yes | Yes | Yes | Yes |
| Case25 | Yes | Yes | Yes | Yes | Yes | Yes | No |
| Case26 | Yes | Yes | Yes | Yes | Yes | Yes | Yes |
| Case27 | Yes | Yes | Yes | Yes | Yes | Yes | Yes |
| Case28 | Yes | Yes | Yes | Yes | Yes | No | No |
| Case29 | No | Yes | Yes | Yes | Yes | No | No |
| Case30 | Yes | Yes | Yes | Yes | Yes | Yes | Yes |
| Case31 | Yes | Yes | Yes | Yes | Yes | Yes | Yes |
| Case32 | Yes | Yes | Yes | Yes | Yes | Yes | Yes |
| Case33 | Yes | Yes | Yes | Yes | Yes | Yes | Yes |
| Case34 | No | Yes | Yes | Yes | Yes | Yes | No |
| Case35 | Yes | Yes | Yes | Yes | Yes | Yes | Yes |
| Case36 | Yes | Yes | Yes | Yes | Yes | Yes | Yes |
| Case37 | Yes | Yes | Yes | Yes | Yes | Yes | Yes |
| Case38 | Yes | Yes | Yes | Yes | Yes | Yes | Yes |
| Case39 | Yes | Yes | Yes | Yes | Yes | Yes | Yes |
| Case40 | Yes | Yes | Yes | Yes | Yes | Yes | Yes |
| Case41 | Yes | Yes | Yes | Yes | Yes | Yes | Yes |
| Case42 | Yes | Yes | Yes | Yes | Yes | Yes | No |
| Case43 | Yes | Yes | Yes | Yes | Yes | Yes | Yes |
| Case44 | Yes | Yes | Yes | Yes | Yes | Yes | Yes |
| Case45 | Yes | Yes | Yes | Yes | Yes | Yes | Yes |
| Case46 | Yes | Yes | Yes | Yes | No | Yes | Yes |
| Case47 | Yes | Yes | Yes | Yes | Yes | Yes | Yes |
| Case48 | Yes | Yes | Yes | Yes | Yes | Yes | Yes |
| Case49 | Yes | Yes | Yes | Yes | Yes | Yes | Yes |
| Case50 | Yes | Yes | Yes | Yes | Yes | Yes | Yes |
| Case51 | Yes | Yes | Yes | Yes | Yes | Yes | Yes |
| Case52 | Yes | Yes | Yes | Yes | Yes | Yes | Yes |

CN Chinese, EN English, HEI medical history and chief complaint with eye examination information, PCC photograph with medical history and chief complaint, GPT Generative Pre-trained Transformer.

| **Supplementary Table S3: Misclassified cases in phase two with the GPT-4.0-PCC method.** | | | | | |
| --- | --- | --- | --- | --- | --- |
| **Number** | **Clinical diagnosis** | **GPT-4.0-PCC-CN diagnosis** | **Description of the picture** | **GPT-4.0-PCC-EN diagnosis** | **Description of the picture** |
| case4 | Ruptured globe injury | Corneal foreign bodies and corneal scratches | Based on the photographs and medical record information provided, the following is evident: 1. a foreign body in contact with the cornea is visible in the right eye, which appears to be a clear piece of glass. The conjunctiva over the cornea is reddened, which may be due to an inflammatory response from scratching and/or the presence of a foreign body. 2. the edge of the foreign body does not appear to penetrate the cornea, but is not far from the center of the cornea, which may explain the symptoms of pain and decreased vision. The central region of the cornea is a critical area for vision, and any injury could result in significant visual impairment. 3 The patient also reported photophobia and tearing, which may be a direct result of corneal damage and intraocular inflammation, as these conditions increase intraocular sensitivity and activate the lacrimal glands. | Laceration or penetration of the cornea | The image provided appears to show an eye with a visible foreign body on the cornea. There is a grayish-blue triangular-shaped object that seems to be a piece of glass embedded in the corneal tissue. The surrounding cornea appears clear, but there is evident conjunctival injection (redness), which may indicate inflammation or irritation. Additionally, there is a small, translucent area around the foreign body that may suggest corneal edema. |
| case9 | Ruptured globe injury | Corneal foreign body | Based on the photographs and medical history information you have provided; it is clear that this patient has suffered mechanical trauma to the right eye. The photographs show a visible metallic foreign body in the middle of the right cornea surrounded by mild edema and vascular congestion. As you can see from the photographs, the edges of the foreign body are sharp, and this injury could have resulted in a rupture or perforation of the cornea. | Corneal foreign body | The image shows a right eye with a visible foreign body on the corneal surface, which appears to be a metallic fragment, likely the "iron filings" mentioned in the case history. The cornea surrounding the foreign body is hazy, indicating corneal edema or abrasion. There is hyperemia of the conjunctiva suggestive of irritation and inflammation. |
| case12 | Corneal penetrating injury | Laceration or perforation of the cornea | Based on the photographs and medical history information provided, this image shows the patient's left eye in which the following can be seen: 1. corneal break: a distinct irregular white spot on the cornea, which usually indicates a corneal break or erosion. 2. Iris damage: There is a loss of pigment and irregular shape in the iris area. 3. Anterior chamber hemorrhage: The anterior chamber of the eye (the area between the iris and the cornea) appears to have a pool of blood. 4. Conjunctival congestion: Redness of the white part of the eye with visible blood indicates conjunctival congestion. 5. Abnormal tears: Excessive tear production that may be caused by eye irritation or injury. | Traumatic corneal ulcers | The image shows a left eye with a visibly opaque, grayish lesion in the central cornea with feathery edges, consistent with a corneal ulcer. There is also a significant amount of conjunctival injection (redness), which indicates inflammation. The corneal ulcer appears to be central and large, which would explain the loss of vision and photophobia due to its location and the possible involvement of the visual axis. Tearing could be a response to the irritation and possible pain. |
| case39 | Corneal penetrating injury | Corneal abrasions or tears | This photo shows a right eye suffering from significant eye damage. It can be seen: - Conjunctival congestion: the white part of the eye is filled with blood vessels and shows a red color. - There appears to be trauma or abrasion on the cornea, possibly from a foreign body. - There is a foreign body in the anterior chamber of the eye that looks like a metal or hard object, which may explain blurred vision and eye pain. - Pupillary reaction or morphology may be impaired (cannot be determined with certainty due to resolution limitations of the images). | Corneal foreign body | The provided image of the patient's eye displays the following features: Conjunctival injection: This refers to the redness of the eye, which is seen in the white part, indicating inflammation or irritation. An irregular pupil shape: The pupil is not perfectly round, which may suggest traumatic mydriasis or an iris sphincter tear. Opaque or hazy cornea: The cornea does not appear as clear as expected, which could imply corneal edema or abrasion. Foreign body on the cornea: There appears to be a brown object adherent to the corneal surface, which is likely the foreign object mentioned. Anterior chamber reaction: There may be cells or flares in the anterior chamber, though this cannot be conclusively determined without proper slit-lamp examination. |

CN Chinese, EN English, PCC photograph with medical history and chief complaint, GPT Generative Pre-trained Transformer.
